# The UK COVID-19 contact tracing app as both an emerging technology and public health intervention: The need to consider promissory discourses

**DOI:** 10.1177/13634593211060768

**Published:** 2021-11-23

**Authors:** Gabrielle Samuel, Rosie Sims

**Affiliations:** King’s College London, UK; Graduate Institute of International and Development Studies, Switzerland

**Keywords:** contact tracing app, COVID-19, media, promissory discourses, public health, sociology of expectations

## Abstract

The UK’s National Health Service (NHS) COVID-19 contact tracing app was announced to the British public on 12th April 2020. The UK government endorsed the app as a public health intervention that would improve public health, protect the NHS and ‘save lives’. On 5th May 2020 the technology was released for trial on the Isle of Wight. However, the trial was halted in June 2020, reportedly due to technological issues. The app was later remodelled and launched to the public in September 2020. The rapid development, trial and discontinuation of the app over a short period of a few months meant that the mobilisation and effect of the discourses associated with the app could be traced relatively easily. In this paper we aimed to explore how these discourses were constructed in the media, and their effect on actors – in particular, those who developed and those who trialled the app. Promissory discourses were prevalent, the trajectory of which aligned with theories developed in the sociology of expectations. We describe this trajectory, and then interpret its implications in terms of infectious disease public health practices and responsibilities.

## Introduction

The UK’s National Health Service (NHS) COVID-19 contact tracing app was announced to the British public on 12th April by Matt Hancock, Secretary of State for Health and Social Care, as a digital solution to managing the COVID-19 pandemic. The app was promised to the public as a technology that would be available ‘within weeks’ (Wright and Knowles, 2020), and would protect the NHS and ‘save lives’ (Gould and Lewis, 2020). The purpose of the app, similar to apps being developed in other countries,^1,2^ was to use Bluetooth-based technology to digitally trace individuals likely to have come into contact with those who had reported symptoms of the virus, and request that they self-isolate. Based on scientific modelling evidence (Ferretti et al., 2020), NHSX, the unit tasked with developing the app, started work on the technology at the beginning of March 2020, and on 5th May 2020 the technology was released for trial on the Isle of Wight. Subsequent research suggested the trial to be associated with a marked reduction in the spread of the virus on the island (Kendall et al., 2020). However, the trial was halted in June 2020, reportedly due to technological issues. The app was later remodelled by NHS Test and Trace using an Application Programming Interface (API) provided by Google and Apple,^3^ and launched in England and Wales in September 2020. At the time of writing the app has been downloaded more than 20 million times.

During the development and trial of the app, the way discourses (‘talk and texts’) about the technology were constructed would have played a role in how the app was perceived, not just through what was said about the technology, but also *how* it was said (Hargreaves and Ferguson, 2000). This is because language is not a neutral way of describing the world, but instead plays a key role in constructing social life (Gill, 2000; Hargreaves and Ferguson, 2000).^4^ For example, it is well established that discourses within the news media influence beliefs, attitudes and perceived norms about science and technology; have dramatic influences on behaviours; as well as an effect on science policy (e.g. Nisbet, 2004).^5^ Being attentive to discourses and their effects is therefore crucial to examine how the public and other actors’ views, experiences and decision-making come to be constructed and shaped around these discourses. The rapid development, trial, and eventual discontinuation of the initial NHSX app over a short period of a few months makes it a useful case to explore the mobilisation and effect of discourses associated with the technology. This is because such discourses can be relatively easily traced from the app’s inception to its use.

We aimed to explore how discourses associated with the app were constructed in the news media, as well as the effect of these discourses on actors – in particular, those who developed and those who trialled the app. Our research questions were (a) which discourses were used to frame the app in newspaper articles? (b) How were these discourses framed and subsequently understood by (i) those involved with the development/governance of the app, and (ii) by those trialling the technology? And (c) did differences emerge between these two sets of actors? We conducted an analysis of newspaper articles that reported on the app from its development to its trial, as well as eight interviews with stakeholders involved in the app’s development and/or governance, and 15 interviews with public members who trialled the app on the Isle of Wight. Our conceptual approach was a critical realist stance: we viewed knowledge, understanding and beliefs about the app as socially constructed through discourses. Alongside this, we took a realist approach to ontology, acknowledging that a reality exists independent of these discourses (Bhaskar, 2008). This perspective allowed us to explore how discourses constructed knowledge and beliefs about the app, while at the same time interpreting the implication of these discourses on the reality of public health practices and responsibilities. Our analysis showed a prevalence of promissory discourses, the trajectory of which could be explained via theories developed in the sociology of expectations. In what follows, we briefly introduce the sociology of expectations before presenting our findings and discussing their implications.

### Sociology of expectations

The sociology of expectations scholarship describes how promissory discourses are consistently associated with emerging new health technologies, that is, technologies that are not yet within the clinical/health application realm, and whose benefits are uncertain (Brown, 2003). This literature does not make claims about the technology itself, nor moral statements about the worth of such promises in terms of a technology’s assumed social value. Rather, it explores the nature of the promises, and argues that they can sometimes over-emphasise benefits through ‘breakthrough narratives’, and can hide the often complex and uncertain nature of both the innovation process as well as the implementation of technologies into practice (Fitzgerald, 2014; Pickersgill, 2011, 2016; Samuel et al., 2021a; Will, 2010). Furthermore, rather than just being ‘vacuous hype’ – a by-product of innovation – promissory discourses and the expectations attached to them constitute the innovation process itself; they not only represent beliefs, views or visions, they *do* something – they are part of the world of action (Tutton, 2017). Specifically, sociology of expectations scholars argue that these expectation discourses act performatively in the present. They prompt alliance-building and secure funding for further technological development (Borup et al., 2006; Brown, 2003; Geels and Smit, 2000), or act as an impetus to drive implementation and delivery of technological innovation into practice (Samuel and Farsides, 2017). However, as the innovation pathway continues, elevated expectations often remain unmet due to underlying issues with the innovation and/or implementation process. The communities of promise – originally generated by these discourses to drive innovation and/or implementation – collapse (Brown, 2003; van Lente, 1993). This can sometimes lead to a range of negative unintended consequences (Fortun, 2001; Hilgartner, 2015; Petersen and Krisjansen, 2015), including broken promises and hopes, disillusionment, damaged credibility of innovators and policymakers, and a public mistrust of innovation and science more broadly (Petersen and Krisjansen, 2015; Samuel and Kitzinger, 2013). Such unintended consequences, when present, are often asymmetrical: those in closer proximity to knowledge production (scientists, innovators, policymakers) have more awareness of the uncertainty and complexity of technological innovation. However, because scientists tend to ‘black box’ areas of controversy and uncertainty, ‘those distant from the research front, and thus not exposed to the art and craft of scientific practice, get a view of science relatively free of doubts and uncertainties’ (Collins, 1999: 165; also see MacKenzie, 1998). This social distance leaves a habitable ‘space’ for futuristic expectations and promises to abound (Brown, 2003; Hedgecoe, 2006; Samuel et al., 2021a; van Lente, 2012).

## Methods

### Research context

This paper was part of a wider project that explored the sociology of the UK COVID-19 app, and the interviews and early interview analysis phases took place in this context. Methods are described briefly here. We refer the reader to Samuel et al. (2021b) for more details. Ethics approval was received from King’s College London research ethics office: MRA-19/20-19251.

### Data collection

#### Interviews

In June 2020, before the app trial was halted, fifteen phone/online interviews were conducted with individuals who were over 18 years old, who resided on the Isle of Wight, and who had the option to download the app (Table 1). Recruitment was online and via snowballing. Interviews lasted 16–67 minutes and were recorded. Interviews asked how interviewees sourced information about the app, their decision to download the technology (or not), their experiences of using the app and their perceptions about the benefits and harms, as well as their views more generally about the app and of contact tracing more broadly.

**Table 1.** Categorisation of interviewees’ demographics.

Gender	Age (years)	Highest education level	Employment status
Female (*n* = 8)	18–39 (*n* = 3)	16 years (*n* = 3)	Employed/self-employed (*n* = 7)
Male (*n* = 7)	40–69 (*n* = 10)	18 years (*n* = 2)	Unemployed (*n* = 1)
	70 plus (*n* = 2)	College (*n* = 2)	Furloughed (*n* = 3)
		Undergraduate (*n* = 3)	Retired (*n* = 4)
		Postgraduate (*n* = 5)	

Between June and August 2020, eight phone/online interviews were conducted with representatives of those associated with the app’s development and/or governance. Interviewees were recruited through online email addresses or snowballing, were recorded, and lasted between 57 and 90 minutes. Interviews explored interviewees’ practices, views, beliefs and experiences associated with the app’s development and governance. Due to the politically sensitive context of app development and the need to maintain confidentiality, no further information is provided about interviewees.

#### News articles

**Figure 1.**
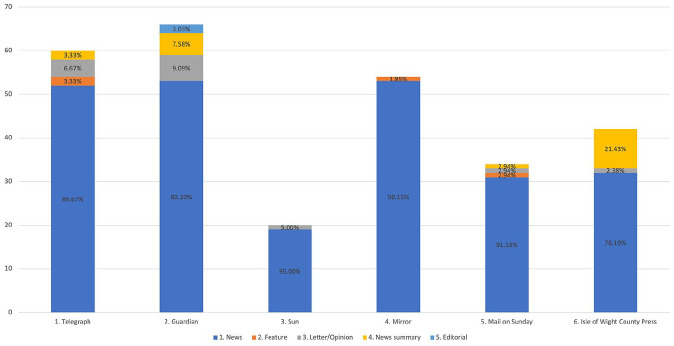
Number of articles reporting on the app per newspaper source, as well as the percentage of the type of article published in each newspaper.

Headlines and lead paragraphs of five UK national newspapers and one local (Isle of Wight) newspaper were searched in Nexis, a comprehensive online news database, on 26th June 2020. The search terms used were: (‘digital tracking’ or ‘digital tracing’ or ‘app’) and (corona* or COVID*) and NHS* (all dates). The Isle of Wight County Press is the main local newspaper on the Isle of Wight, and the only one indexed on Nexis (offline readership of 23,006 copies for a local population of 140,500; plus additional online readership). 62 articles were retrieved/reviewed for a focus on the UK contact tracing app. 42 remained after removing duplicates and non-relevant articles. The five specific national newspapers included the telegraph.co.uk, The Guardian, the MailOnline, the mirror.co.uk and thesun.co.uk. These newspapers included the highest UK readership, offline/online presence, different political leanings (left/right), and different readership (low, mid, high socio-economic status).^6^ One thousand two hundred thirty articles were retrieved/reviewed for a focus on the UK contact tracing app. Two hundred and thirty-four remained after removing duplicates and non-relevant articles. The type of articles and the newspaper within which they were published is shown in Figure 1.

### Data analysis

#### Interviews

Data analysis was conducted via reflexive thematic analysis (Braun and Clarke, 2019). Coding was both deductive and inductive, and conducted by GS and another member of the wider research team. Analysis of the codes was iterative and reflexive, continually going back and forth between the data and theory through a process of constant communication and reflection between wider research team members. During this process, all members of the wider research team read the interview transcripts and discussed findings before, during and after the coding process. Our analysis adhered to the belief that themes ‘do not passively emerge from either data or coding; they are not ‘in’ the data’ (Braun and Clarke, 2019), but rather are actively created as a product of the intersection between our theoretical assumptions, analytic resources, and the data itself. A range of themes were developed around issues of expectations, app governance, trust, and benefits and harms related to the app. This paper focuses only on the theme of expectations. Other themes have been developed elsewhere (Samuel et al., 2021b).

Small modifications to some extracts and the withholding of pseudonym identities in places was required to maintain stakeholder interviewee confidentiality.

#### News articles

A text-only content and discourse analysis was conducted, informed by the methodological approaches of Murdoch and Caulfield (2018), and Nerlich and Halliday (2007). Each of the 234 articles was read in detail to ensure understanding of its context. Articles were coded as previously described (Samuel et al., 2021a). Articles noting benefits but omitting or only briefly mentioning contrasting views (for example, possible harms or technical challenges associated with the implementation of the app) were coded ‘supportive’. Articles noting harms or challenges, but omitting or only briefly mentioning a contrasting view, were coded as ‘critical’. Articles were coded as ‘balanced’ if positive and negative aspects were detailed in equal measure, or if a conclusive stance could not be determined. Coding categories also included publication date, newspaper and type of article.^7^

## Findings

Our findings explore the different phases of promissory discourses associated with the app from its inception and development to its use, and then to the halting of the app trial. In each phase we present the nature of discourses present in the news articles, as well as in the interviews with stakeholders and/or members of the public.

### Developing communities of promise

Following the UK government’s decision to develop the NHS COVID-19 contact tracing app, a steering committee and wider team were selected to start work on the innovation process. Experts were selected for these roles with the prerequisite that they were generally supportive of the UK’s decision. In this way a ‘community of promise’ (Brown, 2003) was formed:

*we also needed to recognise that we were embedded in a program of development so we. . . had to make sure that people could be constructive in terms of pushing the agenda forward in a critical way without derailing it* (stakeholder 1).

Within this community of promise, interviewees described how the app was framed by the government as a technological solution that would function to contain the spread of the virus; ‘at one point [the government said] we are going to build this and it’s going to be perfect’ (stakeholder 7):

*at the beginning Matt Hancock was just dead enthused about apps because. . . he was Mr Digital. And there was the Singapore app and the South Korea app, and they were almost going* “oh the apps are going to save the world” (identity withheld).

Stakeholder 8 explained how these promises were not vacuous hype, but an aspect of the professional vision (Goodwin, 1994) of those who were responsible for the UK’s response to the pandemic, and for developing the app. For this interviewee, these individuals needed to believe and become champions of these promises in order to incentivise the app’s development. This interviewee described this as ‘incentive bias’:

*there is also an incentive bias, right, if you are responsible for part of the* UK’s *response to this thing you want to have tools that are going to really help you. So, you are incentivised to believe the best possible outcome*.

**Figure 2.**
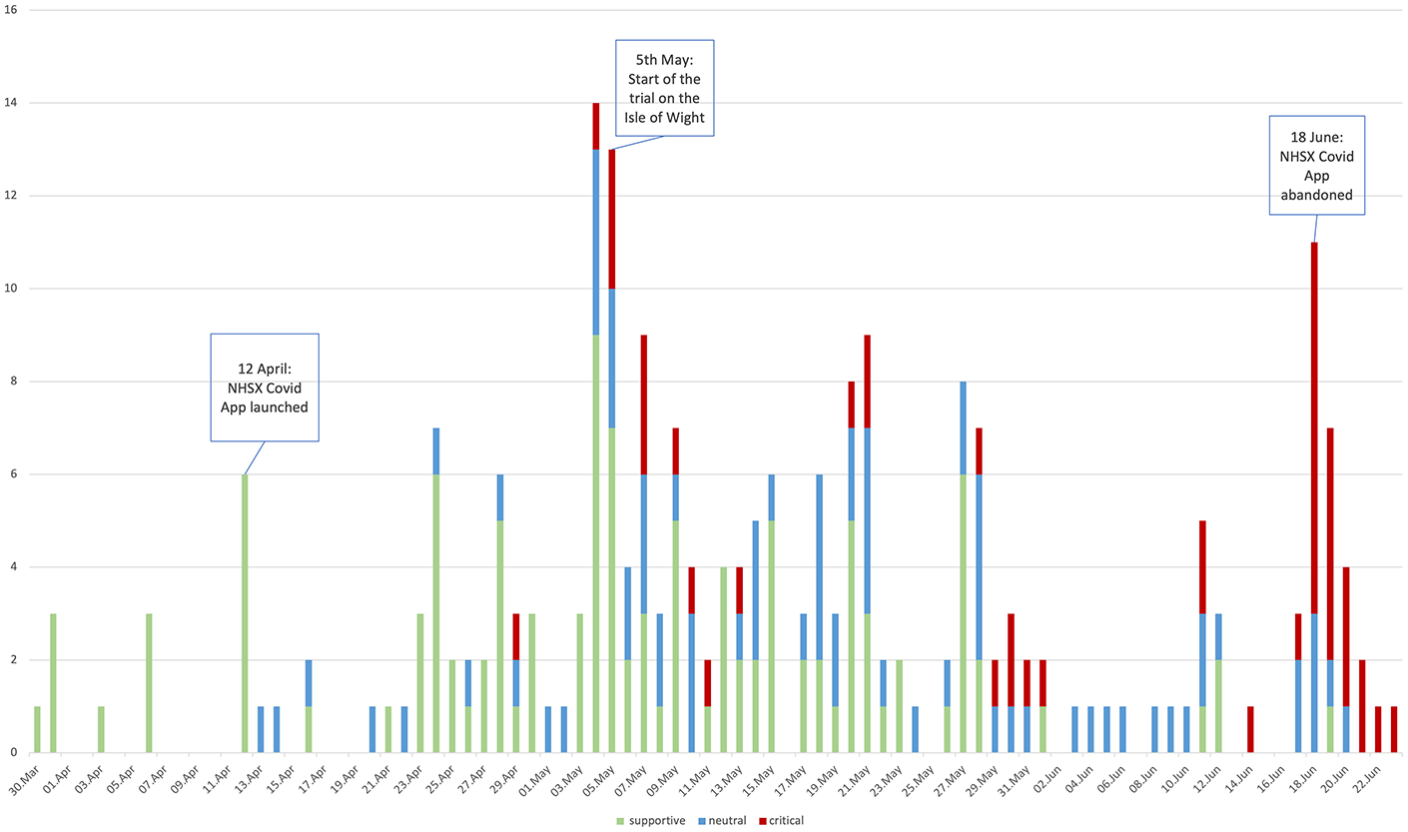
Timeline of discourses concerning the app in national newspapers by date and number of articles.

These promises were also present in the news articles we analysed.^8^ Of the 234 national newspaper articles analysed, about half of the articles portrayed the app in a positive light (48%), a third were neutral in portrayal (32%) and a fifth (19%) were critical or unsupportive. Analysis of the timeline along which national articles were published showed that those articles which were supportive of the app were generally published from the announcement of the app, leading up to and during the beginning of the trial on the Isle of Wight (Figure 2). Many of these articles contained promissory statements, including presenting the app as ‘pioneering’ (The Sun, 4 May 2020), and suggesting ‘the technology could significantly slow the rate of transmission and help countries to emerge from lockdowns safely’ (the Mirror, 31 March 2020).

#### Personal expectations and hidden complexities of the app

Within the community of promise, interviewees adopted different levels of personal expectations compared to the high collective expectations. Only the personal expectations of a small minority – particularly those who, as described above, played a central role in the development of the app (and therefore who could be argued to have the role of incentivising the innovation process) – aligned with those of the collective community (‘and then finally there is this question of whether it is a silver bullet or not. I am still an optimist and think it could have made a huge difference’ (identification withheld)). Such optimism was perhaps associated with the success of using contact tracing apps as seen in other countries, such as China, South Korea and Singapore.

Most interviewees had more sober expectations, understanding the capabilities of the app, the political and social landscape of promissory discourses, and the complexity of the technological innovation process. These interviewees attempted to manage expectations about the app by pointing to the app’s limitations – that the app could never act as a technological solution to containing the pandemic, and that it was always supposed to be one specific aspect of the wider response to the contact tracing programme. In fact, these interviewees were all too aware of the difficulties involved in the app’s development, and highlighted what seemed to be an incredibly complex innovation process fraught with a range of issues. Interviewee 4, for example, spoke about technical issues (how app developers were working on a project that was using Bluetooth as the underlying measuring mechanism – a function that ‘is not designed for measuring distance’), and issues with field tests, which were described as ‘an entire industry in itself’. Other interviewees spoke about the ‘political complexity’ of developing the app (stakeholder 2). Others described how those with different types of expertise – whether that be medical, public health safety, technical/engineering or security/privacy-based – often came into conflict, because they each had different end goals in terms of what aspects of app development should be prioritised (privacy versus safety versus saving lives etc). This was especially the case because the innovation process was taking place within a pressured, politically charged environment and with a huge amount of public and governmental attention. This all stood to illustrate how those involved in the development of the app were aware of the complicated techno-socio-political climate within which innovation was occurring (Hoeyer, 2012).

### Commencing the trial: Promises and a sense of pride

**Figure 3.**
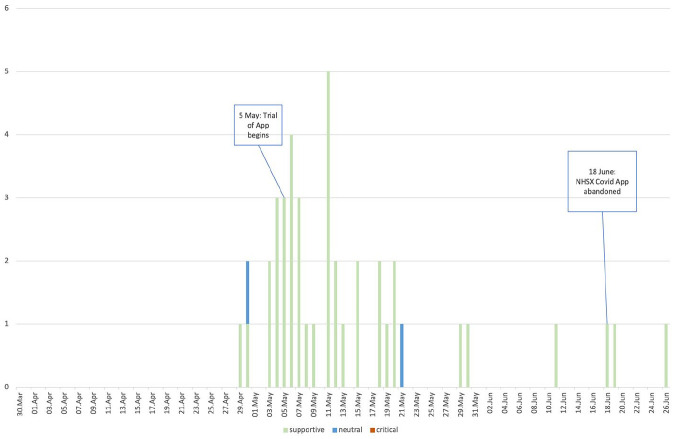
Timeline of discourses concerning the app in the Isle of Wight County Press by date and number of articles.

Despite the complexities associated with the app’s development, the technology was released for trial on the Isle of Wight on 5th May 2020. The Isle of Wight County Press articles were even more supportive of the app than national newspaper articles (95% of articles were ‘supportive’; Figure 3). We identified both promissory discourses – including statements claiming the app will ‘play a vital role in getting Britain back on her feet’ (Isle of Wight County Press, 5 May 2020), and that the app could impact coronavirus to the extent of ‘possibly even eradicating it altogether’ (Isle of Wight County Press, 4 May 2020) – as well as nationalistic discourses explicitly encouraging app downloads by promoting the Island as leading Britain’s way. Local pride was evoked and drawn upon in multiple articles, including statements such as: ‘Islanders are currently the only people in the UK to have this access and continue to lead the way’ (Isle of Wight County Press, 30 May 2020). Other articles celebrated ‘the Isle of Wight blazing a trail for the rest of the country’ (Isle of Wight County Press, 6 May 2020), crowned by reports of a special edition T-Shirt being made with the slogan ‘where the Isle of Wight leads, Britain follows’. References were made to the numbers of current app downloads, and the local MP (Bob Seely, who supported the app trial, and whose presence in the local media was dominating) was sourced as stating; ‘we need to keep downloading the app, making sure it is on, keep sending feedback on the feedback form and by doing that, help the rest of the UK to support the NHS – and care homes – and to stay healthy’ (15 May 2020).

Public interviewees reflected upon these discourses of nationalism, promises to lead the way and an expectation of success.^9^ Interviewee 1 remembered:

*I think it was Boris Johnson who mentioned that it was going to be happening on the Isle of Wight. We were going to be the first area to supposedly lead the way with the new app tracing scheme* (interviewee 1).

When interviewees were asked whether they knew about apps developed in other countries, interviewee 14 explained that while they were not aware of any specifically, ‘I do know there are other ones out there because I do know at the briefing they said that they were trying to make our one the best in the world’. It was perhaps because of these types of discourses that some interviewees described the trial on the Isle of Wight as a ‘brilliant opportunity’ for the Island (‘I thought it was an amazing idea and what a brilliant opportunity’ (interviewee 3)). In fact, this marketing campaign was seemingly aimed at directly targeting the pride residents felt towards the Island in order to encourage them to download the app. As interviewee 5 explained; ‘they were saying it would be a good thing for the Island. I don’t know why they thought it would be good for the Island other than it could contain it [the virus] if it did become a real bad problem on the Island’.

Interviewees seemed to at least initially engage with these aforementioned discourses; at the time of interview, all but two interviewees reported to be supportive of the app, and had downloaded the technology and installed it on their phone.

### Continuing the trial: Towards the ‘cherry on the cake’

While nearly all Isle of Wight interviewees were supportive of the technology, the app did not always live up to interviewees’ expectations. Based on their own experiences, interviewees spoke about both not understanding the technology, as well as having issues with the app’s technical functionality (not being able to upload test results, the app draining their battery life, not alerting interviewees even when household members tested positive for COVID-19, etc.). Interviewees also seemed frustrated with some of the messages provided by the app. Interviewee 7 explained:

*when I got the ping, it just said* ‘stay alert’, *I could still go to the shops, I could still do everything, it just said* ‘stay alert’. *So, if I have potentially got [coronavirus], I* don’t *really want to be going to the supermarket*. . .

Interviewees described forgetting or purposefully choosing to not take their phone out with them, or not keeping it close to their bodies (e.g. leaving it in a supermarket trolley) (interviewees 2, 7, 12). Finally, there was a sense that some interviewees did not understand how the app functioned. For example, interviewee 5 was confused about how the app notified an individual of a potential virus exposure even though that individual had not been out for a few days: ‘how can that be, because they haven’t been anywhere’.^10^

Stakeholder interviewees who were close to the innovation process were unconcerned about these issues. They already understood the limitations of the app, and emphasised that the technical issues were a normal aspect of any new app. They explained that such issues would ordinarily be later updated in subsequent versions (stakeholder 6). Several Isle of Wight interviewees agreed. For example, interviewee 15, who had previously worked within the government, seemed to be aware of the complexity that surrounded innovations. Interviewee 8 was also understanding of the emerging technical difficulties, describing them as part of any ‘trial and error’ process, as well as the fact that the point of the trial was to determine whether the app worked or not; ‘like all these things, it’s trial and error. It’s something we didn’t know anything about, and you have got to try something to see if it works’. However, nearly all other Isle of Wight interviewees spoke passionately about their disappointment related to these issues, which were entangled with their hopes and high expectations about the technology. Interviewee 10 explained, ‘I was expecting more from the app’ and interviewees described themselves ‘losing faith’ in the app (interviewee 1) and being ‘disappointed’ – ‘I was disappointed that the app was not working on a lot of phones, not taking off in the way that I had hoped’ (interviewee 11). Interviewee 7 spoke about being ‘completely put off’ following an incident in which her sister had reported symptoms through the app, but that did not lead to his own phone ‘pinging’ even though they lived together. Interviewee 5, who was initially happy to trial the app, now expressed disenchantment, viewing the technology as ‘a total waste of time’:

I’m *seeing hundreds of people every day and not once has it gone off.. . .[. . .]. . .I* can’t *believe* I’ve *seen that amount of people. . .and not one. . .*I’ve *become very disenchanted with it. . .*I’m *thinking* I’m *going to delete. . .[. . .]. . .*it’s *a total waste of time* (interviewee 5).

### From hype to ‘experimental’: Bursting the hype bubble

The decision was made by the UK government in late June to halt the trial.^11^ Prior to this decision, the government changed the way it framed the app from a promise that the technology would ‘save lives’ to an app that was better viewed as ‘experimental’: ‘at various times it was backtracked on and. . .then, by the end, it was “this is very experimental”‘(stakeholder 7). Stakeholder 3 reflected on how the government used the metaphors of the app being ‘the cherry on the cake’ to disseminate this change of discourse. Stakeholder interviewees perceived the reasons for this change in discourse differently. Some attributed it to the government listening to interviewees’ own protests that the app would never be a technological solution to contact tracing, and this needed to be made clear to the public. Other interviewees pointed to an attempt made by the government to provide a discourse that would give permission for them to end the trial. This is because while technical issues are tricky to justify within promissory discourses, they are more easily justified when associated with an experimental technology. Here then, the government was seen as ’managing’ or ’governing’ expectations in light of the technological issues (Hielscher and Kivimaa, 2019), shifting the initial hype into a downward trajectory. This down-grading reflected a newer understanding that the app’s future would never become that which was originally promised, and was now more aligned with those interviewees who initially had more sober expectations of the app. Even stakeholder 4, who initially had high expectations about the app, reflected on the government’s promise that the app would be ready ‘in a few weeks’, in light of their evolving exposure to the various complexities involved in developing the app:

*you have a concept of an app and you think* that’s *a great idea. . .but then when it comes down to it, there are all these tricky complicated components that we* don’t *really understand. How much foresight was there to recognise [this]?. . .It was launched as something that would be ready in a few weeks. . ..we are all learning as we go along because* it’s *a new experimental technique*.

Such views of the app and the bursting of the hype bubble were also reflected in the national news articles analysed, which started to include overtly critical/negative discourses from the app’s launch onwards, intensifying around the time the trial ended (Figure 2). Discourses included marking the app as a failure and a waste of public resources, and that despite scrapping the current version of the NHS app altogether, the government showed no accountability nor recognition of this. Some sources were quoted as describing Island residents as being the government’s ‘guinea pigs’ (Telegraph, 21 April 2020; Guardian, 4 May 2020; MailOnline, 28 May 2020) or ‘lab rats for a costly experiment’ (Isle of Wight County Press, 18 June 2020). Having said this, even when the trial had been halted, media discourse in the Isle of Wight local paper was still positive (in terms of the trial having revealed improvements to be made, lessons learned and so forth).

### Ending the trial: Public interviewees’ perspectives and the forgotten Island

In spite of the Isle of Wight interviewees’ perceptions about the limitations of the app, interviewees spoke about their disappointment that the government had ended the trial. Part of this sense of disappointment seemed intricately tied to the way in which the government had communicated this decision: interviewees drew on the same discourses and wording used by the government (particularly around no longer being ‘a priority’) in their interviews. Interviewee 5, for example, emphasised a sense of ‘being forgotten’ as the government re-prioritised their efforts on a different app:

I’ve *seen bits and pieces today that have said* it’s *no longer a priority. . .their priority is [now] more the track and trace one that they are using now on the mainland. It just feels* “oh well, it’s the Island”, *and we’ve been forgotten*. . .it’s “oh it didn’t work over there but we’re just, you know, just wipe it aside and carry on with something else” (interviewee 5).

Interviewee 16 was similarly deeply offended to no longer be seen as ‘a priority’. For them, it would have been polite, given they were involved in app trial, to have been provided with some more information about the progress and resultant termination of the trial:

*if the app has been a waste of time, then just let us know*, don’t *say* it’s *not a priority. Because* that’s *a bit of a smack in the face for us because [we are] the people who are trying it,. . ..Normally if you ask somebody very kindly please take part in something, then you keep them appraised of the situation, and the progress or otherwise* (interviewee 16).

Finally, other interviewees, who had very much been affected by the government’s original framing of the app as ‘leading the way’, described how their sense of residential pride in the Island – and of being involved with the trial – had been taken away from them:

*Those of us on the Isle of Wight now feel a bit stupid because it was trumpeted as* “the Isle of Wight leads the way”. *And* let’s *face it, people tend to ignore the Isle of Wight mostly, and we all felt* “oh well, great we are doing something really good”. *And it turned out not to be the case. So*, it’s *made an awful lot of people very, very cynical on the Island* (interviewee 10).

## Discussion

Many of the hallmarks of the promissory discourses so well studied in the emerging technologies literature were evident in our findings – both when considering the COVID-19 app as an emerging innovation, as well as during the trial in its role as a public health practice.

The trajectory of the promissory discourses (hype cycle of expectations) aligned with theories developed in the sociology of expectations, and these theories can guide us to the potential benefits and harms that can emerge from the use of such discourses (Petersen and Krisjansen, 2015). On the one hand, future-making using positive expectations is performative – it drives innovation by developing alliances and rallying resources (Borup et al., 2006; Brown, 2003; Geels and Smit, 2000), or, as we have shown here in the case of the contact tracing app, by providing an impetus and incentive to drive the innovation process (Samuel and Farsides, 2017). The large numbers of people dying or predicted to die from COVID-19 at the time the app was developed would have fed into this incentive, and the incentive to try to find alternatives to blanket lockdowns that were harming the economy and infringing people’s liberties.

At the same time, as we also saw in our findings, promissory discourses can lead to unintended consequences in the form of broken hopes and promises, disillusionment and disappointment.^12^ In fact, the sociology of expectations literature reminds us of the intricate relationship between promissory discourses and hope, and of the fact that this hope is linked to trust and belief in promissory discourses (Samuel et al., 2021b; Sheikh and Hoeyer, 2018). In more serious instances, and particularly in instances in which promises are more speculative, more extreme harms can eventuate from too much trust being placed in such promises. For example, Petersen and Krisjansen (2015) describe recent reports of patients having died following speculative stem cell treatments based on high hopes of success. While promises attached to the contact tracing app are less speculative, especially because apps have successfully been used to manage the pandemic in other countries (China, South Korea and so forth), they still had unintended effects. Furthermore, their effects were asymmetrical, dependent on the proximity to the knowledge production process, with those further away from the innovation process (public interviewees) generally having less understanding of the complexities associated with the app technology, and therefore tending to place more faith in the promissory discourses. However, it is precisely these individuals who most need to be informed about the technology. This is because while those close to the knowledge production process (public health experts, researchers, innovators, etc.) can navigate promissory discourses by drawing on a range of experts to help them understand an issue associated with the technology and frame it in a certain way, the public is solely reliant on the communication from those close to technological innovation. Discourses about the app framed in a promissory way were most likely the key source of information about the app for members of the public.^13^ What was required, as we have argued elsewhere (Samuel et al., 2021b), is clear communication about the app’s relative benefits and risks. The public does not necessarily need to be involved with the development of the app (co-production), but communication about the app needed improving. Furthermore, our findings illustrate an asymmetry of expectations and interests between those associated with the development and/or governance of the app and general members of the public, suggesting a need for better communication and transparency at the level of app innovation. Elsewhere, we have explored how poor communication at this level was in part related to the hierarchical governance structure associated with the app’s development process, and discussed different ways in which this structure, and the communication practices within it, could have been improved (Samuel and Lucivero, 2021).

In the literature on emerging technologies, there have been multiple calls for a reworking of the economies of expectation so that the uncertainties known to those closest to knowledge production become more transparent for those more impacted by the broken promises (Brown, 2003; Massarella et al., 2018).^14^ However, from our own brief exploration of the literature, there seems less engagement or questioning of promissory discourses around the implementation of public health practices for infectious diseases (i.e. when the technology is viewed as having high social and health value). It is important to consider what type of discourses are appropriate for dissemination in these circumstances. This is especially true given that in the local Isle of Wight newspaper, promissory discourses were only associated with the public health trial intervention, and did not appear in the newspaper prior to this. Public health practices are increasingly drawing on emerging digital technologies for surveillance and practice, and these increasingly blur the boundaries between technological innovation and public health intervention. As they do, balancing the reliance on promissory discourses for public health practice implementation (Massarella et al., 2018) with potential unintended consequences if expectations are unmet will become more salient. As we saw with the extensive amount of marketing that was a vital aspect of the Isle of Wight trial, the promissory discourses acted performatively by promoting buy-in for pilot projects (Massarella et al., 2018). At the same time, expectations that were misleading or did not align with user experiences of the technology and/or intervention led to disillusionment, and even to a lack of trust (Samuel et al., 2021b). Further research is required to explore issues around promissory discourses associated with public health interventions in detail (including how promises associated with public health practices for infectious diseases develop (by who and how), how they manifest (role of policymakers, newspapers etc), and how they interrelate with wider policy, the government, and other interests of public health experts/surveillance). Further research is also needed to explore whether promises can serve a protective function, especially in regard to initial setbacks surrounding an innovation, and if so, what specific function of expectations may need to be achieved (and in what ways). Nevertheless, from our findings, we can already suggest that where the hype cycle might be problematic is in the lack of transparency and effective communication. For public health practices – and as has been called for previously – justifications should be publicised and open to public scrutiny in order to foster public awareness, confidence, assent, feedback on local conditions, trust, legitimacy and compliance (McDougall, 2010).

Relatedly, alongside promissory discourses, we identified a nationalistic ‘imaginary’ discourse in local news articles that persuaded individuals to participate in the trial by appeals to Island interests, identity, and social obligation (Jasanoff and Kim, 2009). Similar findings have been reported in the biobanking literature, which often points to a drawing on discourses of nationalism, culture and ideology, where loyalty to a particular region is seen as being one element for participation (Busby and Martin, 2006; Tsai and Lee, 2021). Our findings showed how, through the mixture of both promissory discourses and altruistic discourses of solidarity, an imaginary was created that was imbued with implicit understandings of what is good or desirable in the social world (Jasanoff and Kim, 2009). The future-oriented visions and promises attached to the app, along with calls of social obligation, constructed the trial of the app as a venture which was morally good, which was valued because of its ability to bring health benefits, and which was desirable in the social world of the Isle of Wight. In the emerging technologies literature, there have been calls to question what values are hidden when the socio-technical imaginaries developed through these expectations emerge (Jasanoff and Kim, 2009). This suggests a need to extend such questioning to the similar imaginaries constructed by infectious disease public health researchers, and we call for more research in this area.

Furthermore, and finally, in our findings these discourses seemed to feed into an expectation different to the one associated with the app itself – that is, they were associated with expectations of taking part in the trial, and how this was linked to national pride and solidarity. Indeed, much of the disillusionment experienced by interviewees at the end of the trial could perhaps be understood as both related to the app itself, as well as to the way in which their *expectations* of taking part in the trial mapped onto their *experiences* of the trial. This points to the importance of understanding and managing these different types of expectations – and the potential intended and unintended consequences that come from each of them – when considering future digital technologies for public health interventions.

### Limitations

The small sample size of self-selected interviewees is always a limitation of interview studies because it only allows the capture of the views of those who wish to be interviewed. Furthermore, some of our interviews with stakeholders associated with the development and/or governance of the app were retrospective, being conducted after the end of the app trial. It is therefore possible that interviewees’ views about the app were influenced by the benefit of hindsight, therefore shaping more sober expectations about the technology. Having said that, our interviews were both rich and insightful, and analysis suggested interviewees were being open and candid about their personal views and experiences. Nonetheless, it is likely that interviews could have represented a form of intervention, prompting deeper reflection about the app.

Our media analysis included coding of the newspaper articles as promissory/supportive, neutral or critical. Our discourse analysis provided examples of these discourses. A more in-depth discourse analysis would have given a deeper understanding of how these discourses were constructed. Furthermore, our analysis only included newspapers rather than broader media/social media. If time and resources would have allowed, analysis of the latter would have been preferable. This is because, while newspapers represent a key source of news information, social media is also a prominent news source. Exploring how, if at all, discourses associated with the app differed between these two news sources could have informed our understanding of the relationship between how discourses are portrayed in newspapers and social media, and if different or particular discourses are more prominent in one more than another.

Our analysis ended when the trial of the app was halted. The aim was to continue the analysis longitudinally for 3 months after the trial (follow-up interviews with the public, longer news article analysis). However, at the time the trial was halted, there was political uncertainty about when the new app would be released at all, and so a decision was made to end data collection at the end of June 2020. It would have been useful and interesting to understand how expectations were adjusted and mobilised at the launch of the new app after the ‘trough of disillusionment’, though a lack of time and resources prohibited this.

Finally, we address the limitations of choosing the lens of expectation theory. We are aware that expectation theory offers just one framing of technological innovation (Sovacool and Hess, 2017), and can be deemed simplistic given that the role of promissory discourses and expectations in the innovation process will ultimately depend on their interrelation with other discourses (e.g. negative, or critical discourses, social media, other stakeholders) and factors (technological, economic, political factors and values, etc.) in the social-technological innovation network (Porter and Randalls, 2014; Thyroff et al., 2018). Indeed, the development of the NHSX app followed a very bumpy road to innovation that was steeped in controversy in terms of its ethical legitimacy, technical feasibility and reliability. In particular, scholars, other commentators and the media not only heavily criticised the government’s choice of app design, which was perceived to overly intrude on the right to privacy (Anderson, 2020; Lucivero et al., 2020; Sharon, 2020), but also the governance structure established to oversee the technology’s development, as well as the openness and transparency with which this governance process was communicated to the public (AdaLovelace Institute, 2020; Lucivero et al., 2020; NHSX, 2020; Parker et al., 2020).^15^ A focus on expectations and promises, then, hides the role of these other factors and discourses in the innovation process.^16^ However, it was not our aim to explore the innovation process here. Rather, we wished to focus on how socio-technical change associated with the app was intimately tied to expectations about it (Sovacool and Hess, 2017). The sociology of expectations offered a useful way to understand this. Finally, we are also aware of the absence of conceptual space in this scholarship regarding whether a technology will live up to its promise – while most technologies will not, some inevitably will, and this is not considered in this literature.

In conclusion, we have shown how promissory discourses, the hallmark of emerging technologies, were evident in the public health practice of surveillance, mediated through the app. We have drawn on the sociology of expectations literature to help understand these discourses, as well as responsibilities attached to them. More research is needed to fully understand the implications of promissory discourses in the public health arena.

## Notes

1.
https://www.top10vpn.com/research/investigations/covid-19-digital-rights-tracker/

2. In fact, many apps developed in other countries, for example France and Norway, were based on science published by UK app team.

3. Application Programming Interface: a standard to interact with particular software features and services, allowing modular development and interoperability between devices and apps.

4. This is also the case for our own language.

5. One of the most widely cited examples of news media influence on health decisions is the measles mumps and rubella vaccine (MMR) controversy – though the media attention surrounding the bovine spongiform encephalopathy (BSE) and genetically modified (GM) crops controversies offer equally impressive examples.

6. See https://www.pressgazette.co.uk/uk-newspaper-and-website-readership-2018-pamco/; https://en.wikipedia.org/wiki/List_of_newspapers_in_the_United_Kingdom_by_circulation; https://www.thestudentroom.co.uk/showthread.php?t=1562617; https://www.statista.com/statistics/246077/reach-of-selected-national-newspapers-in-the-uk/.

7. Further coding categories were applied (Samuel and Lucivero, 2021; Samuel et al., 2021b; *forthcoming*), but not relevant here.

8. And supported by an unpublished grey literature analysis, which illustrated how documents produced by the government, parliamentary committees and NHSX tended to more prominently note the app’s benefits compared to documents published by non-government independent commentators.

9. See https://www.iow.nhs.uk/news/CORONAVIRUS-TEST-TRACK-AND-TRACE-PLAN-LAUNCHED-ON-ISLE-OF-WIGHT.htm for how the NHS communicated news about the trial.

10. The delay relates to the time between exposure to the individual who had contracted the virus, and said individual displaying symptoms and/or receiving a positive test result.

11. Discussing why the trial was halted is beyond the focus of this paper. However, our interviews suggested that technical issues were compounded by public attention/criticism of the app (in terms of legitimacy), as well as other political factors.

12. This may have been particularly relevant here given the nature of the pandemic and the strain on people’s mental wellbeing.

13. The public did have other sources of (non-expert) information, most prominently, social media (Samuel et al., 2021b).

14. Such calls have also been made repeatedly in the public understanding of science literature.

15. For more detail/references, see (Samuel and Lucivero, 2021).

16. For example, the black-boxing of uncertainty around technologies may not only provide explanatory power for expectation theories, but may also relate to fears around negative media reporting and the need to maintain public trust.
